# Bioactive nutraceuticals oligo-lactic acid and fermented soy extract alleviate cognitive decline in mice in part *via* anti-neuroinflammation and modulation of gut microbiota

**DOI:** 10.3389/fnut.2023.1116278

**Published:** 2023-03-09

**Authors:** Hamid M. Abdolmaleky, Yin Sheng, Jin-Rong Zhou

**Affiliations:** Nutrition/Metabolism Laboratory, Department of Surgery, Beth Israel Deaconess Medical Center, Harvard Medical School, Boston, MA, United States

**Keywords:** cognition, nutraceuticals, oligo-lactic acid, fermented soy extract, neuroinflammation, microbiota, gene expression

## Abstract

**Introduction:**

Cognition decline is associated with aging and certain diseases, such as neurodegenerative or neuropsychiatric disorders, diabetes and chronic kidney disease. Inflammation/neuroinflammation is considered an important causal factor, and experimental evidence suggests that anti-inflammatory natural compounds may effectively prevent cognitive decline. The goal of this study was to evaluate the effects of two natural bioactive agents, oligo-lactic acid (LAP) and fermented soy extract (ImmunBalance, IMB), on cognition in an adenine-induced cognitive impairment mouse model and to investigate the modulation of related biomarkers.

**Methods:**

Male C57 black mice were randomly assigned into the following experimental groups and received the corresponding treatments for 2 weeks before the use of adenine for model development: (1) negative control; (2) model control: injection of adenine at 50 mg/kg daily for 4 weeks; (3, 4) IMB groups: adenine injection and IMB oral gavage at 250 and 1,000 mg/kg BW, respectively; and (5) LAP group: adenine injection and LAP oral gavage at 1,000 mg/kg BW. One week after the model was developed, mice were evaluated for cognitive performances by using Y maze test, novel object recognition test, open field test, and Barnes maze tests. At the end of the experiment, brain tissues and cecum fecal samples were collected for analysis of gene expression and gut microbiota.

**Results:**

Mice treated with LAP or IMB had significantly improved spatial working memory, spatial recognition memory (LAP only), novel object recognition, and spatial learning and memory, compared with those in the model group. Gene expression analysis showed that, among a panel of cognition related genes, six of them (ELOVL2, GLUT4, Nestein, SNCA, TGFB1, and TGFB2) were significantly altered in the model group. LAP treatment significantly reversed expression levels of inflammatory/neuroinflammatory genes (SNCA, TGFB1), and IMB significantly reversed expression levels of genes related to inflammation/neuroinflammation, neurogenesis, and energy metabolism (ELOVL2, GLUT4, Nestin, TGFB1, and TGFB2). The altered microbiome was attenuated only by IMB.

**Discussion:**

In conclusion, our data showed that LAP improved cognition associated with regulating biomarkers related to neuroinflammation and energy metabolism, whereas IMB improved cognition associated with regulating biomarkers related to neuroinflammation, energy metabolism, and neurogenesis, and modulating gut microbiota. Our results suggest that LAP and IMB may improve cognitive performance in mice *via* distinct mechanisms of action.

## Introduction

Cognition is regarded as a higher function of the central nervous system (CNS) and includes attention, learning, memory, and executive processes (1). Cognition decline is associated with aging and certain diseases, such as neurodegenerative or neuropsychiatric disorders, diabetes, and chronic kidney disorder (CKD) (2). Despite efforts to develop novel therapeutic agents to improve cognitive function, few viable treatment options exist. However, several lines of evidence indicate that natural bioactive agents may provide effective preventive and/or therapeutic strategies to delay cognitive decline (3). More recent research data also provided strong evidence that nutritional compounds not only mediate their effects though their natural bioactive agents, their influence on gut microbiome alterations may also have significant beneficial or harmful consequences (4).

The science of microbial colonization of gut was limited before the development of microbial 16S rRNAs sequencing technology. Now, it has been shown that the mammals gut contains diverse bacterial elements which their total number is 10 times more than their body cells. Surprisingly, the gut microbial population collectively contain 100 times more genes than human which are involved in the digestion of many nutritional compounds or production of nutrients and vitamins (Reviewed in Alam et al. (4)). There is also evidence that nutritional elements and food compounds affect the gut microbiome composition and thus their function as well. For example, it has been shown that in mice fed with high-fat diet, silybin decreases *Firmicutes*, *Lactobacillus*, *Lachnoclostridium*, and *Lachnospiraceae_UCG-006*, and increases the gut level of *Bacteroidetes*, *Bacteroides*, *Blautia*, and *Akkermansia* (5), while epigallocatechin-3-gallate (EGCG) increases *Verrucomicrobia*, *Enterococcaceae*, and *Verrucomicrobiaceae*, but decreases *Deferribacteres*, *Lachnospiraceae*, *Desulfovibrionaceae*, *Bacteroidaceae*, *Proteobacteria*, *Prevotellaceae*, *Deferribacteraceae*, and *Rikenellaceae* (6).

Furthermore, it has been shown that gut microbiota not only plays an important role in normal brain development, but also has significant impacts on animal behavior and cognitive status. For example, metabolomic analysis showed that four metabolites linked to neuropsychiatric disorders were down-regulated in germ-free mice (7). Additionally, gut microbiota manipulation in germ-free mice impacts fear extinction learning that is associated with gene expression alterations in single-nucleus RNA sequencing analysis of glia, excitatory neurons, and other brain cells in the medial prefrontal cortex (7). These mice also exhibit postsynaptic dendritic spines remodeling and hypoactivity of cue-encoding neurons in transcranial two-photon imaging analysis. However, selective microbiota re-establishment could restore normal extinction learning (7). Notably, increases in plasma tryptophan and hippocampal concentration of serotonin and its metabolite (5-hydroxyindoleacetic acid) was also shown in germ-free mice compared to the control mice. While the brain neurochemical changes remained stable until adulthood, restoring microbial colonization post weaning could reverse behavioral alterations in affected mice (8).

It has been reported that mice treated with adenine showed depressed locomotor activity associated with cognitive impairment, depleted brain norepinephrine, dopamine and serotonin, disrupted blood–brain barrier, and increased brain inflammation (9–12). In our previous study applying this mouse model to investigate the effect of bioactive components on CKD (13), we found that the adenine-treated mice had increased inflammation in circulation and in kidney tissues. Bioactive components, an oligo-lactic acid product (LAP) and/or a fermented soy extract (ImmunBalance, IMB), alleviated adenine-induced CKD associated with decreased circulating inflammatory cytokines and tissue inflammation. Our preliminary observation also indicated that mice in the model group had cognitive problems. Interestingly, LAP- and IMB-treated groups showed improved cognition and were more energetic and healthier than positive control mice (adenine treated). These results and observations suggest that LAP and/or IMB may have beneficial effects on improving cognition/alleviating inflammation-associated cognitive impairment.

In this study, we propose to investigate the effects of LAP and/or IMB on improving cognition in adenine-induced mouse model of cognitive impairment, and to determine if improved cognition is associated with modulation of gut microbiota using and expression of inflammation related genes in brain tissue.

## Materials and methods

### Materials

A soy extract, IMB was prepared by a koji fermentation of defatted soybeans with *Aspergillus oryzae* and lactic acid bacteria (*Pediococcus parvulus* and *Enterococcus faecium*) according to a proprietary fermentation technology, followed by water extraction and purification of Koji polysaccharides®. IMB was provided by Nichimo Biotics Co., Ltd., Japan. Oligo-lactic acid product (LAP) was a condensate of about nine ester-linked molecules of L-lactic acid that was purified from fermentation products of sugar beet and corn with *Lactobacilli* according to a proprietary process. LAP was provided by LifeTrade Co., Ltd., Japan.

### Animal study

**Figure 1:**
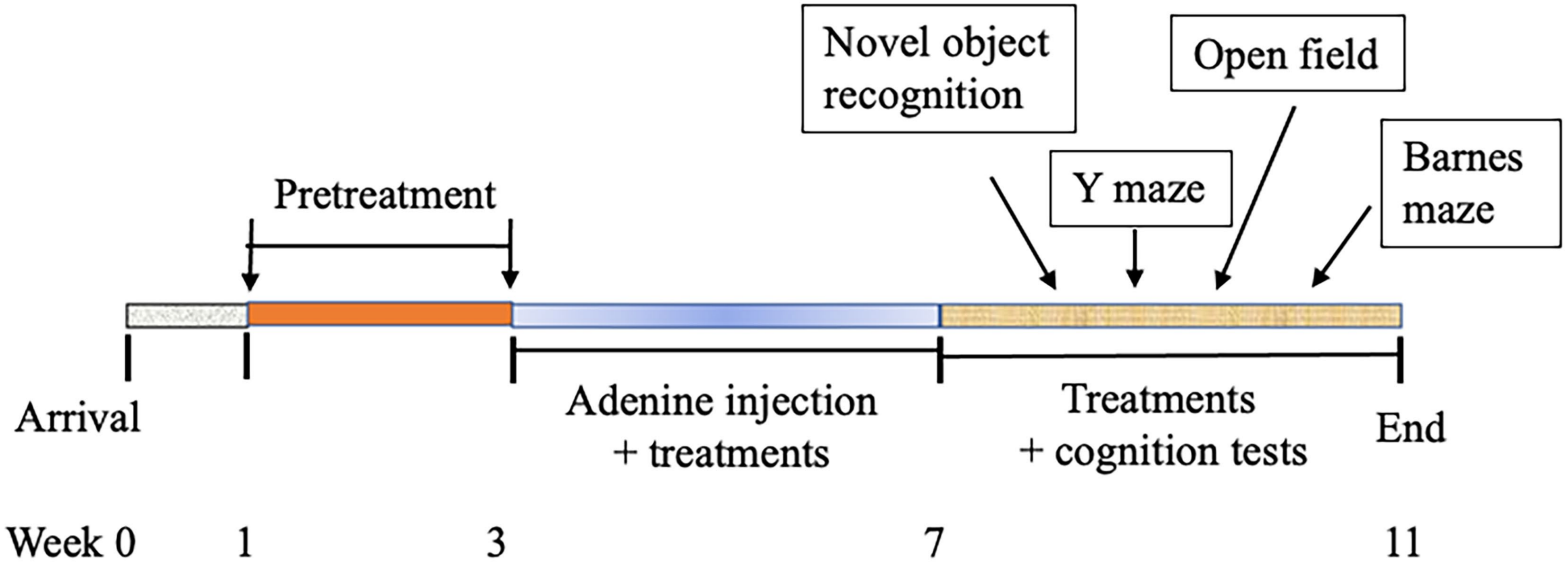
Diagram of experimental schedule.

Male C57BL/6 mice (8–9 weeks of age) were purchased from Taconic (Germantown, NY, United States), housed in a room at a temperature of 22 ± 2°C, relative humidity of about 60%, with a 12 h light–dark cycle, and free access to an AIN-93 M diet. After an acclimatization period of 1 week, mice were randomly assigned into the following five groups (*n* = 8/group) and receive the corresponding treatment for 2 weeks before the use of adenine for model development: (1) negative control (NC): PBS injection and PBS oral gavage daily; (2) model control (MC): adenine injection intraperitoneally (i.p.) at a dose of 50 mg/kg daily for 28 days and PBS oral gavage daily; (3) IMB-low treatment (IMB-L): adenine injection, oral gavage of IMB at 250 mg/kg BW; (4) IMB-high treatment (IMB-H): adenine injection, oral gavage of IMB at the 1,000 mg/kg BW; and (5) LAP treatment: adenine injection, oral gavage of LAP at 1,000 mg/kg BW. Our previous animal study indicated that IMB had a dose-dependent effect, whereas LAP did not show clear dose-dependent effect (13). Therefore, we used two doses of IMB (250 and 1,000 mg/kg BW) and one dose of LAP (1,000 mg/kg BW) in this study to evaluate the effect of treatments on cognition improvement and biomarkers alterations. Body weight and food intake were measured weekly. The cognition/memory evaluations were started 1 week after adenine injection was finished. A diagram of experimental protocol is included as Figure 1.

### Methods for memory/cognition tests

#### Y maze test

The Y maze was used to measure spatial working and recognition memory by making use of a rodent’s natural exploratory instincts (14). The Y-maze consists of three arms of equal length interconnected at 120°. The Y Maze test includes two sessions. The first session measures spatial working memory, and the second session measures spatial recognition memory. The experimental procedures are described as follows:

*Spatial working memory test:* During the first session of the experiment to measure spatial working memory, all three arms of the maze were open. Specifically, mice were placed onto the end of one arm and allowed to explore freely for 5 min. The sequences of the arms entries were recorded. The spontaneous alternation behavior was calculated as the number of triads (three different arms, ABC not ABB) that contain entries into all three arms divided by the total visits. The first session represents a classic spontaneous alternation test of spatial working memory.

*Spatial recognition memory test:* The second session measures spatial recognition memory. During the testing phase, one of the arms of the maze was blocked while the mouse was allowed for a 5-min exploration of only two arms of the maze. After a 30 min break, the partition was removed and the mouse was allowed for another 5-min exploration. Since the first 2 min of activities were the most sensitive to measure spatial preference for a new arm, the time and number of visits to the new arm were calculated for the first 2 min of testing. The first 5 min of activities were also recorded and the motor activity was calculated as the number of arms visited for the whole period of the test. This experiment takes advantage of the innate tendency of mice to explore novel unexplored areas (e.g., the previously blocked arm). Mice with intact recognition memory prefer to explore a novel arm over the familiar arms, whereas mice with impaired spatial recognition memory enter all arms randomly. Thus, this experiment represents a classic test for spatial recognition memory.

Data were recorded and analyzed by using the Smartsuper software (Harvard Apparatus, Holliston, MA, United States) for the time spent in each arm overall, and the time spent in each arm for the first 2 and 5 min.

#### Novel object recognition test

This test was used to examine memory in animals. The mouse was presented with an object during acclimation and then in testing presented with a novel (new) object, and the amount of time the animal spends exploring the novel object was recorded. The mouse was then challenged further with a subsequent test where the novel object was moved. Here instead of a three-chamber apparatus, which was used in previous experiments (thus mice were familiar with), a rectangle chamber was used. The experimental protocols are described as follows:

*Acclimation*: Each animal was placed in a rectangle chamber without objects and was allowed to explore for 10 min. The procedures were repeated 24 and 48 h later, thus the total acclimation was 3 days.

*Experiment setup-day 4:* In the rectangle chamber apparatus, two identical objects were placed in the test chamber. The animal activity was recorded to track how much time was spent to interact with each of the objects, the “right object” and “left object,” for a fixed session length of 10 min. The animal’s nose must be within 1 cm of the object and directed at the object, or actually touch the object in order to be considered time spent exploring the object. After 10 min, we removed the animal was putted back in its home cage and the chamber and objects were cleaned with 70% ethanol to remove any smell.

*Experiment setup-day 5*: One old object and one novel (new) object were putted in the chamber, and the animal activity was recorded for 5 min instead of 10 min. After an intertrial interval (ITI) of approximately 2 h, the location of the old object was moved so that it was located on a different wall and the steps were repeated, again using a 5 min trial length.

#### Open field test

Open field test was used to evaluate mouse fear and anxiety levels. In this test, a mouse was placed in rectangle apparatus and its activity was recorded for 10 min. Mouse instinct drives it to search in the corners and angles of the chamber instead of the middle part (open field) of the chamber. In this test, we also placed two identical objects in the chamber, one in a corner and another one in the middle of the chamber to assess the frequency and time the mouse spent with each object. The mouse was expected to spend more time with the object placed in the corner than that in the middle.

#### Barnes maze for spatial learning and memory test

Mice spatial learning and memory were assessed using Barnes maze test (Harvard Apparatus). Barnes maze is one of the best and ideal apparatus for testing spatial learning and memory in mice (14, 15). The Barnes maze motivates animals to hide from the bright, open platform by finding the small, dark “escape box.” Animals learn the escape box’s location without the stress of swimming or food deprivation, and false escape boxes remove the possibility of inadvertent cues. With a variety of color options, Barnes maze adjusts to fit a wide range of experiments. In Barnes test, mice are trained in 3–5 sessions to find the escape box and after 5–7 days mice are tested for the time spent to find the escape box. We tested the mice for two successive days after 6 days interval. The data were collected and analyzed using the Smartsuper software (Harvard Apparatus).

At the end of Barnes maze test, mice were sacrificed, and biological samples (blood, brain, and cecum) were collected for analyses. A piece of mouse brain sample from the left frontal lobe (~30 mg) was used for RNA and DNA extraction. The fecal sample of mouse cecum was used for DNA extraction and microbiome profiling.

### Gene expression analysis by quantitative real-time PCR (qRT-PCR)

An aliquot of hippocampus-containing brain tissues was used for gene expression analysis. In brief, following RNA extraction using Direct-Zol DNA/RNA MiniPrep kit (Zymo Research, Irvine, CA, United States) and cDNA synthesis using Bio-Rad iScript cDNA synthesis kit (Hercules, CA, United States), expression levels of genes related to neurotransmission, inflammation, metabolism, and neuronal protection/regeneration were determined using CFX384 Touch real-time PCR detection system and *Power* SYBR™ Green PCR Master Mix (Applied Biosystems, Bedford, MA, United States) by following the established procedures (13). These genes included brain-derived neurotrophic factor (BDNF), dopamine beta-hydroxylase (DBH), dopamine receptor D2 (DRD2), ELOVL fatty acid elongase 2 (ELOVL2), glucose transporter 4 (GLUT4), Huntington-associated protein 1 (HAP1), 5-hydroxytryptamine receptor 2A (HTR2A), interleukin 1β (IL1B), IL4, IL6, NESTIN, phosphoinositide 3-kinase (PI3K), solute carrier family 6 member 4 (SLC6A4), synuclein alpha (SNCA), transforming growth factor beta 1 (TGFB1), TGFB2, and TSC complex subunit 1 (TSC1). All mRNA quantification data were calculated using the 2^∆∆*C*t^ method and normalized to GAPDH, presented as folds to the negative control (NC). The primer sequences of genes were taken from Harvard Primer Bank (listed in Table 1) designed for real-time PCR analysis and tested to assure that they generated a single PCR product.

**Table 1:** The primer sequences for qRT-PCR.

Genes	Forward	Reverse
BDNF	TCATACTTCGGTTGCATGAAGG	AGACCTCTCGAACCTGCCC
DBH	GAGGCGGCTTCCATGTACG	TCCAGGGGGATGTGGTAGG
DRD2	ACCTGTCCTGGTACGATGATG	GCATGGCATAGTAGTTGTAGTGG
ELOVL2	CCTGCTCTCGATATGGCTGG	AAGAAGTGTGATTGCGAGGTTAT
GAPDH	AGGTCGGTGTGAACGGATTTG	GGGGTCGTTGATGGCAACA
GLUT-4	ATCATCCGGAACCTGGAGG	CGGTCAGGCGCTTTAGACTC
HAP1	AGGTGAACCTGCGAGATGAC	TGCTGGTCTTGATCCCTCTGT
HTR2A	TAATGCAATTAGGTGACGACTCG	GCAGGAGAGGTTGGTTCTGTTT
IL1B	GCAACTGTTCCTGAACTCAACT	TGGATGCTCTCATCAGGACAG
IL4	GGTCTCAACCCCCAGCTAGT	GCCGATGATCTCTCTCAAGTGAT
IL6	CCAAGAGGTGAGTGCTTCCC	CTGTTGTTCAGACTCTCTCCCT
Nestin	CCCTGAAGTCGAGGAGCTG	CTGCTGCACCTCTAAGCGA
PI3K	TTATTGAACCAGTAGGCAACCG	GCTATGAGGCGAGTTGAGATCC
SLC6A4	GTCATTGGCTATGCCGTGGA	CACCCATTTCGGTGGTACTG
SNCA	GACAAAAGAGGGTGTTCTCTATGTAG	GCTCCTCCAACATTTGTCACTT
TGFB1	CTCCCGTGGCTTCTAGTGC	GCCTTAGTTTGGACAGGATCTG
TGFB2	CTTCGACGTGACAGACGCT	TTCGCTTTTATTCGGGATGATGT
TSC1	ATGGCCCAGTTAGCCAACATT	CAGAATTGAGGGACTCCTTGAAG

### Microbiome analysis of cecum fecal samples

DNA was extracted using Quick-DNA Fecal/Soil Microbiome Miniprep kit (Zymo research, cat # D6010) from 30 mg of fecal sample from the mouse cecum for microbiota analysis. After quantitation and quality control using NanoDrop One (Thermo Scientific), the extracted DNA (each ~100 ng/μl, in total volume of 100 μl) was diluted (10 ng/μl) and subjected to examination with one set of universal and one set of Lactobacillus specific primers (Table 2) (16) to reconfirm DNA quality. Then, DNA samples were sent to CoreBiome, Inc.^1^ for microbiome profiling using amplicon sequencing targeting variable region 4 of the bacterial 16S ribosomal RNA gene (16S rRNA). All quality control measures by CoreBiome experts confirmed the reliability of data presented in the result section.

**Table 2:** Primer sequences of microbiota analysis.

Lac-F	AGCAGTAGGGAATCTTCCA	*Lactobacillus* genus
Lac-R	CACCGCTACACATGGAG	*Lactobacillus* genus
Uni331F	TCCTACGGGAGGCAGCAGT	All bacteria
Uni797R	GGACTACCAGGGTATCTATCCTGTT	All bacteria

The details of the methods are presented in www.corebiome.com and elsewhere by the developers (17).

### Statistical analysis

Data were expressed as the group mean ± standard error and analyzed by one-way analysis of variance (ANOVA) test, followed by multiple comparison of least-significant difference (equal variances assumed) or Dunnett’s T3-test (equal variances not assumed) to evaluate the difference of parametric samples among groups. A *p*-value of <0.05 was considered statistically significant.

## Results

### Effects of treatments on body weight and food intake

**Figure 2:**
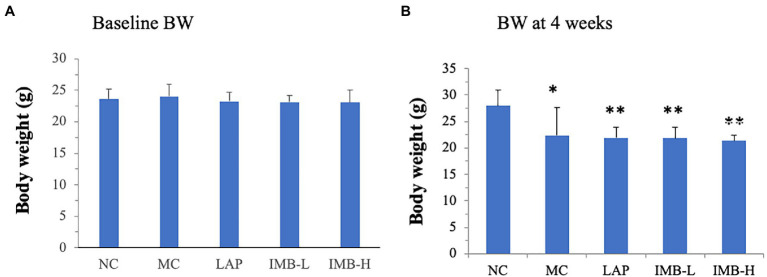
Effects of treatments on body weights (BW). **(A)** Baseline BW; **(B)** BW after 4 weeks of model induction. Values are expressed as mean ± standard error. Within each panel, values with a superscript symbol are significantly different from the NC. *p < 0.05; **p < 0.01.

There was no significant difference in the baseline body weight among experimental groups (Figure 2A). As expected, model induction *via* daily adenine injection reduced body weight (Figure 2B) in all modeled groups, compared with the negative control group, whereas there were no significant differences of body weight among the modeled groups. Similarly, mice in the modeled groups reduced food intake, compared with the NC group, but there were no significant differences of food intake among the modeled groups (data not shown).

### Effects of LAP and IMB treatments on spatial working memory and recognition memory

**Figure 3:**
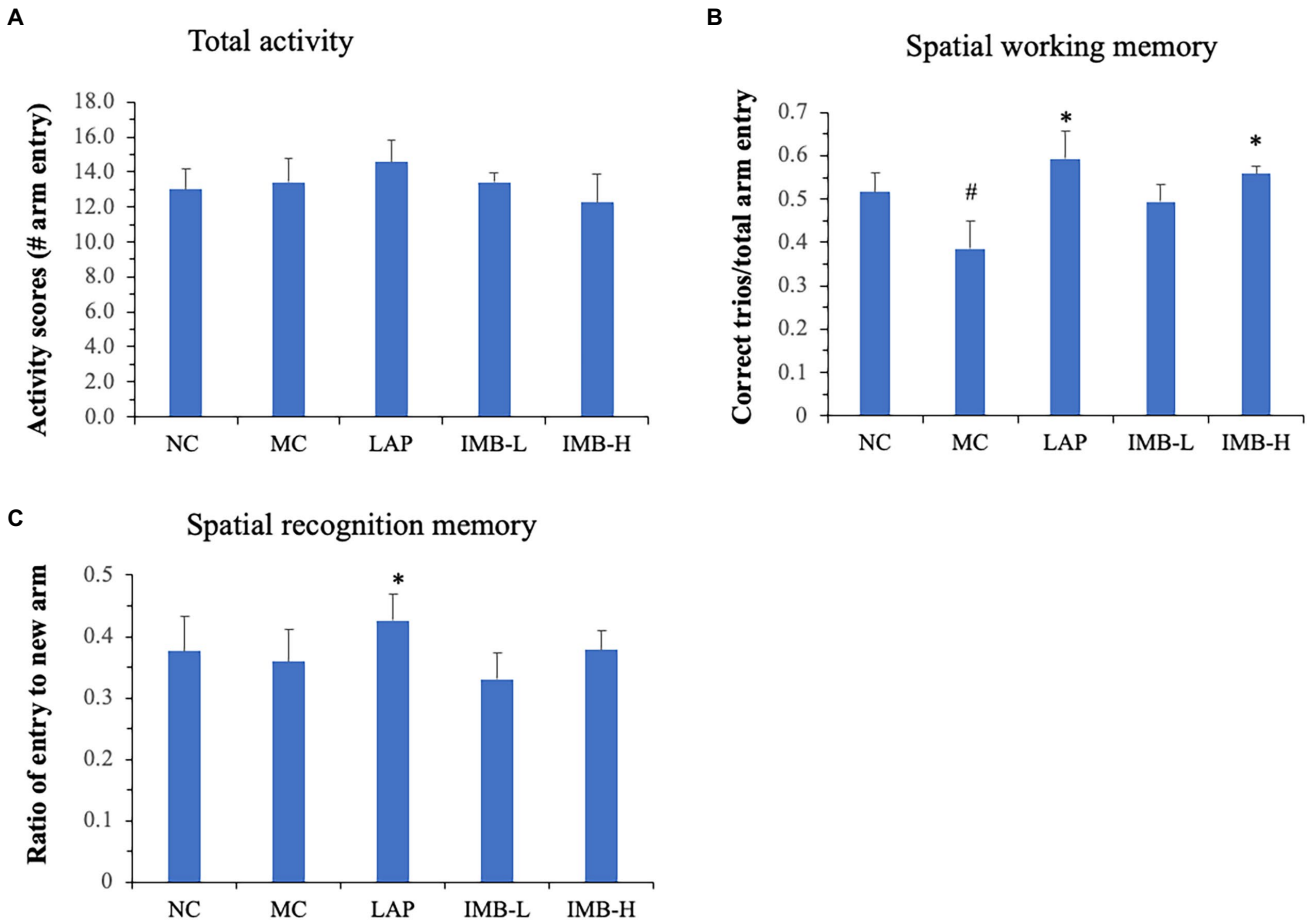
Effects of treatments on spatial working memory and spatial recognition memory, as measured by Y maze test. **(A)** Total activity measured as the total numbers of arm entries in 5 min; **(B)** Spatial working memory measured as the correct trios (ABC or ACB) vs. total arm entry in 5 min; **(C)** Spatial recognition memory measured as the average number of entries to new arm in 5 min. Values are expressed as mean ± SE (*n* = 8). Within each panel, values with a superscript symbol “*” are significantly different from that of the MC. *p < 0.05.

Spatial working memory and recognition memory tests were performed using Y maze as described in methods. There were no significant changes in the total activity (numbers of arm entry) among different groups (Figure 3A). The model development significantly impaired spatial working memory (Figure 3B, *p* < 0.05) but did not significantly affect recognition memory (Figure 3C). Compared with the MC, mice treated with LAP had significantly better spatial working memory (Figure 3B, *p* < 0.05) and recognition memory (Figure 3C, *p* < 0.05). IMB treatment improved spatial working memory in a dose-dependent manner and mice treated with the high dose IMB had significantly increased spatial working memory (Figure 3B, *p* < 0.05).

### Effects of LAP and IMB treatments on novel object recognition and spatial learning and memory

**Figure 4:**
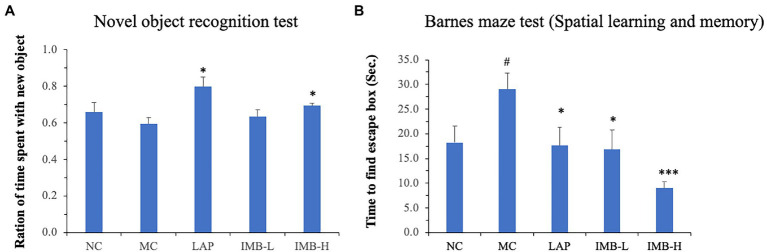
Effects of treatments on novel object recognition memory and spatial learning and memory. Mouse novel object recognition memory was tested 24 h after training **(A)**; mouse spatial learning and memory was evaluated in Barnes maze test **(B)**. Values are expressed as mean ± SE (*n* = 7–8). Within each panel, values with a superscript symbol “*” is significantly different from that of the MC (*p < 0.05; ***p < 0.001); and the value in the MC with a superscript symbol “#” is significantly different from that of the NC (^#^*p* < 0.05).

As shown in Figure 4A, the MC mice had non-significant impairment of novel object recognition memory; mice treated with LAP significantly improved novel object recognition by over 30% (*p* < 0.05). IMB showed a dose-dependent effect on novel object recognition memory, and the high dose IMB treatment showed a significant effect (*p* < 0.05).

Barnes maze test was applied to determine spatial learning and memory. Compared with the NC, mice in the MC showed significantly impaired spatial learning and memory (Figure 4B, *p* < 0.05). This impaired memory was significantly improved by the treatment of LAP (*p* < 0.05) or IMB (*p* < 0.05 for IMB-L; *p* < 0.001 for IMB-H). Overall, IMB treatment showed a suggestive dose-dependent effect.

The results of open field test did not show significant alterations among any experimental groups (data not shown).

### Effects of LAP and IMB treatments on gene expression levels of related biomarkers in brain tissues

**Figure 5:**
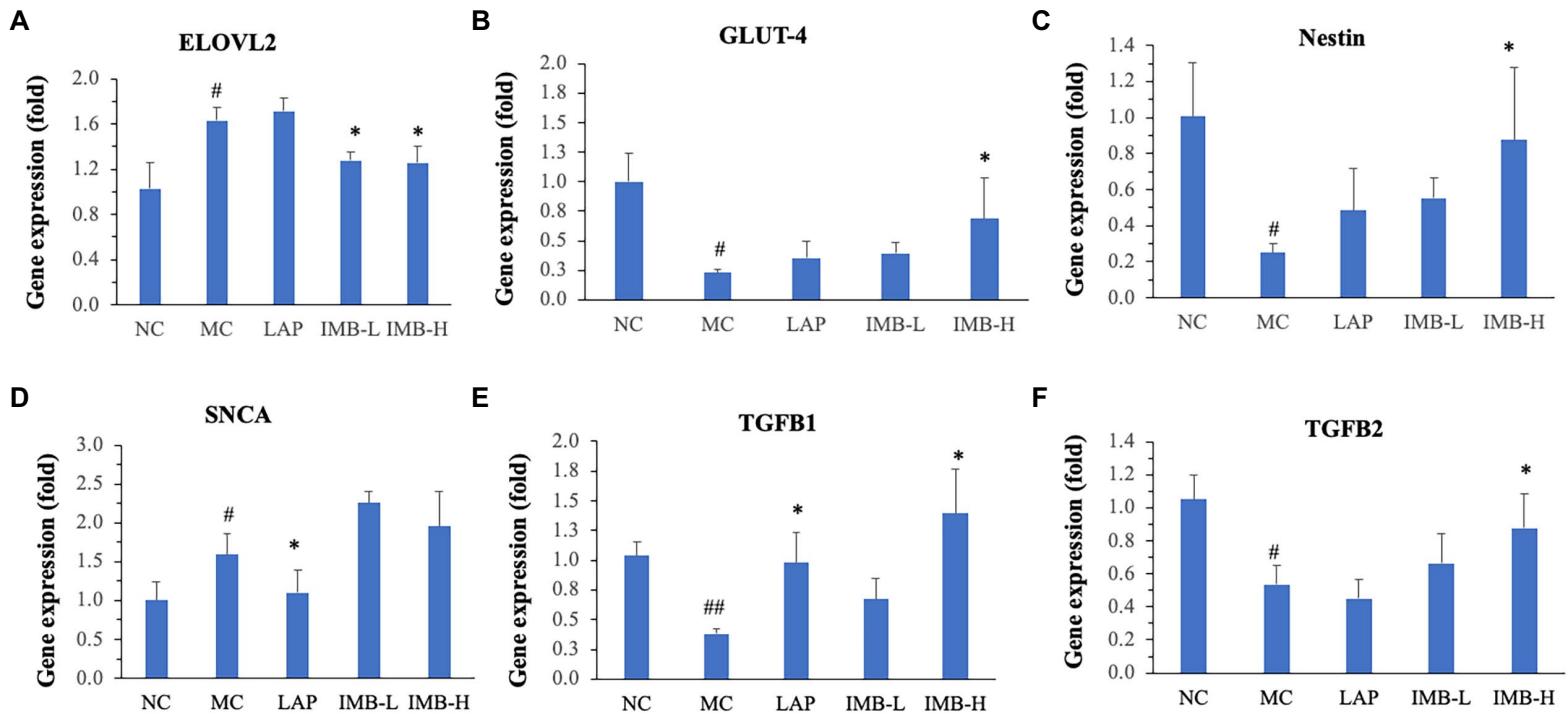
Effects of treatments on expression levels of genes that were significantly altered by model development and recovered by LAP and/or IMB treatment. ELOVL2 **(A)**, GLUT-4 **(B)**, Nestin **(C)**, SNCA **(D)**, TGFB1 **(E)**, and TGFB2 **(F)**. Values are expressed as mean ± SE (*n* = 7–8). Within each panel, values with a superscript symbol “*” are significantly different from that of the MC (*p < 0.05; **p < 0.01); and the value of the MC with a superscript symbol “#” is significantly different from that of the NC (^#^*p* < 0.05; ^##^*p* < 0.01).

We explored the expression levels of a panel of genes related to neurotransmission, inflammation, metabolism, and neuronal regeneration at RNA levels (including, BDNF, DBH, DRD2, ELOVL2, GLUT4, HAP1, HTR2A, IL1B, IL4, IL6, NESTIN, PI3K, SLC6A4, SNCA, TGFB1, TGFB2, and TSC1) aiming to identify candidate genes whose expressions were significantly altered in the model group and were significantly reversed by LAP and/or IMB treatments. Six candidate genes (ELOVL2, GLUT4, SNCA, Nestein, TGFB1, and TGFB2) were identified. As shown in Figure 5, compared with the NC, the model development (MC) significantly decreased the expression levels of GLUT4, NESTIN, TGFB1, and TGFB2 genes by 75, 80, 60, and 50%, respectively (*p* at least <0.05), and significantly increased the expression levels of ELOVL2 and SNCA genes by 60 and 55%, respectively (*p* < 0.05). Expression of TGFB1 was significantly increased by LAP (>100%, *p* < 0.05) and IMB-H (>150%, *p* < 0.05; Figure 5E); the decreased expression of TGFB2 in model mice trended to be normalized by IMB treatment (Figure 5F). The reduced expression levels of GLUT4 (Figure 5B) and NESTIN (Figure 5C) in the MC mice were increased significantly by IMB-H treatment (>100 and 150%, respectively, *p* < 0.05). The increased expression of ELOVL2 in the MC mice was reversed only by IMB-L or IMB-H treatment (Figure 5A, ~40%, *p* < 0.05), whereas the increased expression of SNCA in the MC mice was significantly altered by the LAP treatments (Figure 5D, *p* < 0.05).

### Effects of LAP and IMB treatments on gut microbiota

**Figure 6:**
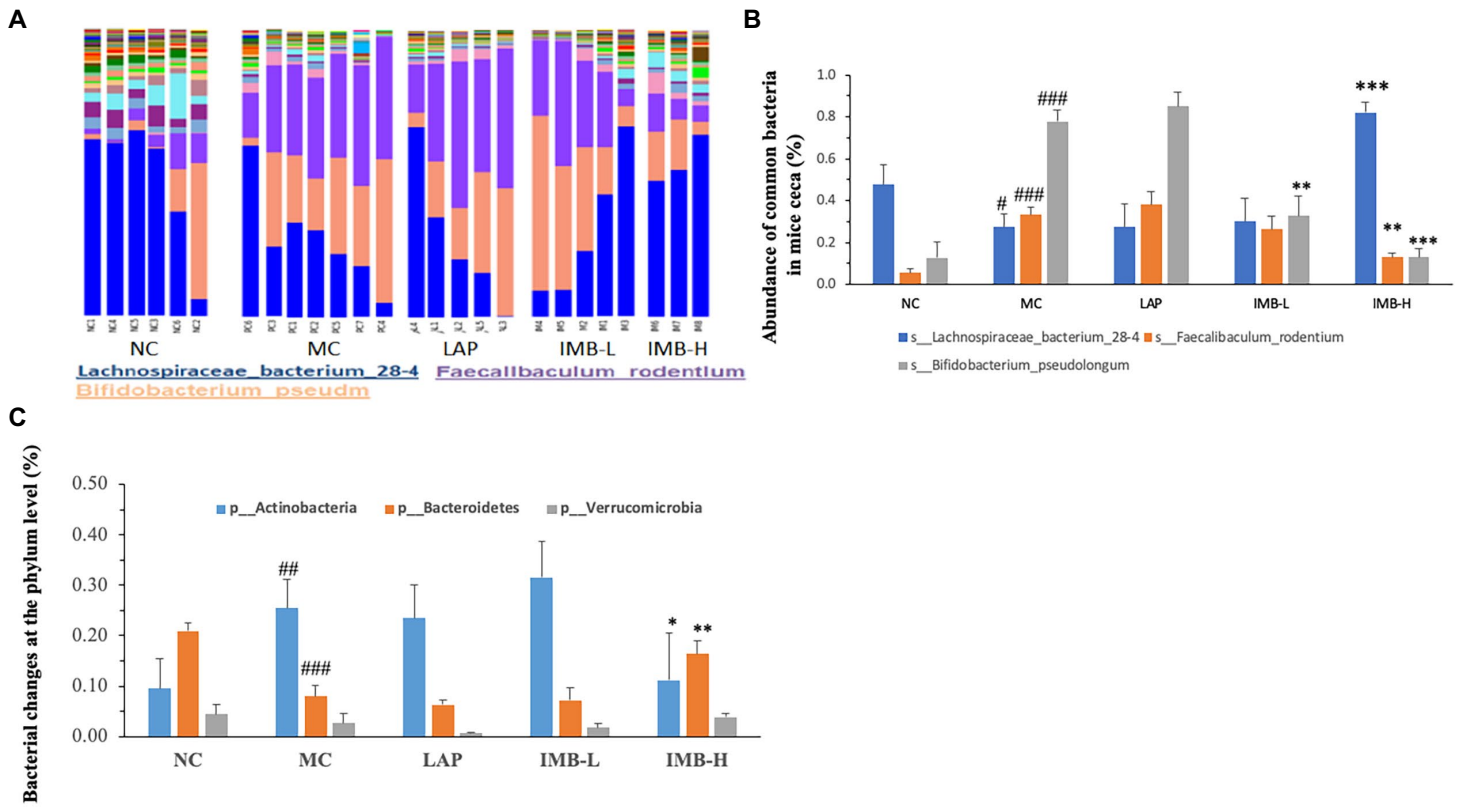
Effects of treatments on gut microbiota. **(A)**. A portray of cecum bacterial changes; **(B)**. Effects of treatments on the abundance of three common bacteria; **(C)**. Effects of treatments on the abundance of three phyla. Values are expressed as mean ± SE (*n* = 3–7). Within each panel **(B or C)**, values with a superscript symbol “*” are significantly different from that of the corresponding MC (*p < 0.05; **p < 0.01; ***p < 0.001); and the value of the MC with a superscript symbol “#” is significantly different from that of the corresponding NC (^#^*p* < 0.05; ^##^*p* < 0.01; ^###^*p* < 0.001).

In general, the abundance of almost 60% (30 out of 51) of bacterial species exhibited significant changes in the MC, compared with that in the NC. As shown in Figure 6A the diversity of bacterial species decreased in the MC group which was recovered in part by IMB-H. Figure 6B shows the abundances of three common bacteria that were significantly altered in the MC group and were also reversed/normalized in the IMB-H group. Compared with the NC, model development (MC) significantly decreased the abundance of *Lachnospiraceae bacterium* 28-4 (*p* < 0.05), but significantly increased abundances of *Bifidobacterium pseudolongum* and *Faecalibaculum rodentium* (*p* < 0.001). In general, IMB treatments recovered aberrant alterations in a dose-dependent manner and the high dose IMB (IMB-H) treatment had significant effects. On the other hand, LAP treatment did not show significant effects on reversing the abundance of these common bacteria altered in the MC group (Figure 6B).

The abundances of two phyla (out of seven phyla) were also altered significantly in the MC group (Figure 6C). Compared with the NC, the MC significantly increased the abundance of *Actinobacteria* (*p* < 0.01) and significantly decreased the abundance of *Bacteroidetes* (*p* < 0.001) (Figure 6C). Those alterations were significantly reversed by the treatment of high dose IMB (IMB-H), but not LAP or IMB-L.

**Figure 7:**
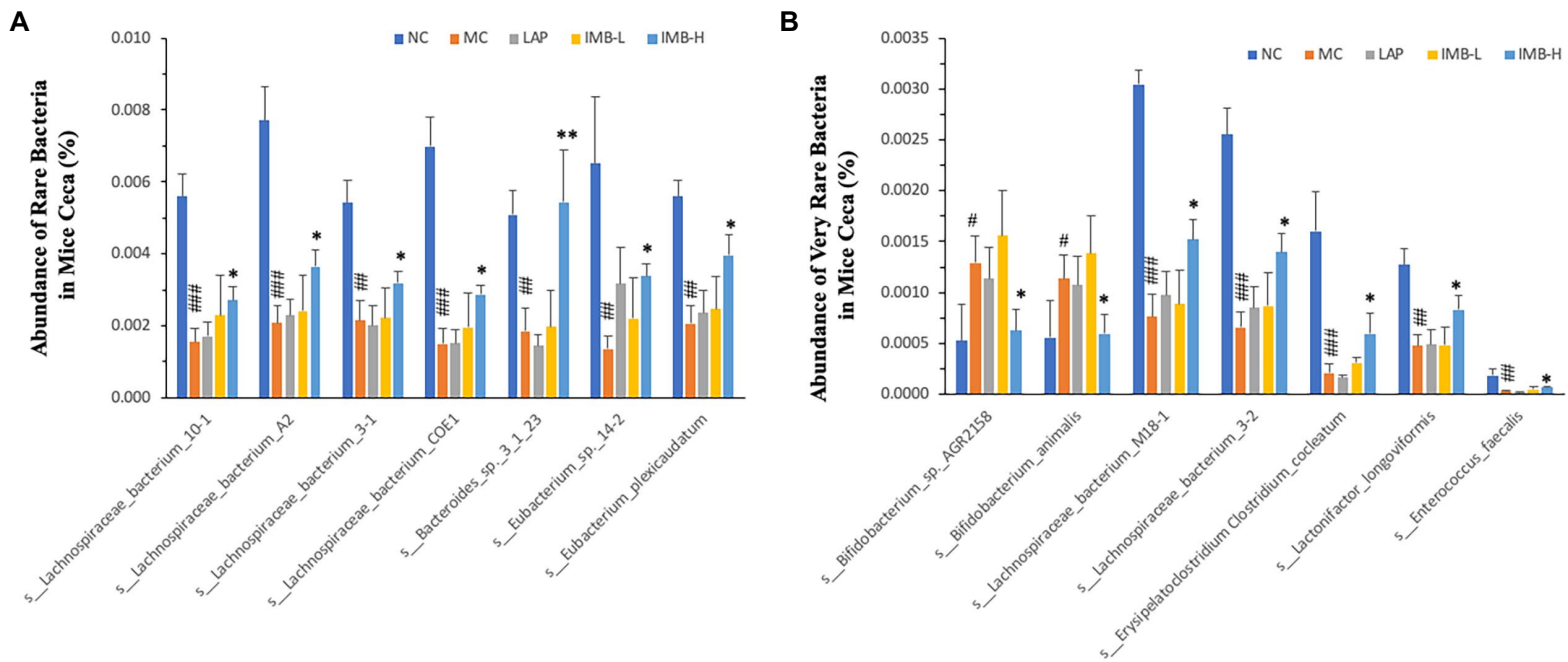
Effects of treatments on abundance of rare **(A)** and very rare **(B)** bacteria in mice ceca. Within each panel, values with a superscript symbol “*” are significantly different from that of the corresponding MC (*p < 0.05; **p < 0.01; ***p < 0.001); and the value of the MC with a superscript symbol “#” is significantly different from that of the corresponding NC (^#^*p* < 0.05; ^##^*p* < 0.01; ^###^*p* < 0.001).

We also found that the model development significantly altered the abundances of several rare (Figure 7A) and very rare (Figure 7B) bacteria, and some treatments significantly reversed these alterations. In general, IMB treatment showed a dose-dependent effect on reversing model-induced alterations and the high dose IMB treatment effects were statistically significant. On the other hand, LAP treatment did not show significant effect on the abundances of those bacteria.

## Discussion

In this study, we evaluated the effects of two nutraceutical components, LAP and IMB, on cognitive status of adenine-induced cognitive impairment mouse model. Mice treated with LAP had significantly improved spatial working memory (Figure 3B), spatial recognition memory (Figure 3C), novel object recognition (Figure 4A), and spatial learning and memory (Figure 4B), compared with those in the MC group. Similarly, IMB treatment significantly improved spatial working memory (Figure 3B), novel object recognition (Figure 4A), and spatial learning and memory (Figure 4B) in a dose-dependent manner. Gene expression analysis showed that, among a panel of genes, six of them (ELOVL2, GLUT4, Nestein, SNCA, TGFB1, and TGFB2) were significantly altered in the model group, and their expression levels were significantly reversed by LAP (SNCA and TGFB1) and/or IMB (ELOVL2, GLUT4, Nestin, TGFB1, and TGFB2). On the other hand, the altered microbiome was attenuated only by IMB-H.

Our study showed that LAP could significantly alleviate adenine-induced cognitive impairment. While the mechanisms of LAP actions may remain elusive, it is possible that reduction of inflammation/neuroinflammation may be one of the important mechanisms. The causal role of inflammation/neuroinflammation in cognitive impairment is well recognized. Our previous study showed that LAP reduced inflammatory lesions in kidney tissues, circulating levels of proinflammatory cytokines (IL-6 and IL-12p70), and mRNA levels of proinflammatory cytokines (IL-6, TLR-4, F4/F80, and IL-1ß) in kidney tissues (13). While histopathological evaluation of inflammation in brain tissues was not performed (in part due to insufficient tissue availability), we found that adenine significantly increased SNCA mRNA level and decreased TGFB1 mRNA level, and these alterations could be significantly reversed by LAP treatment. SNCA has recently been shown to be associated with decline of cognitive functions in older adults including Alzheimer’s disease patients possibly *via* mediation/induction of neuroinflammation (18, 19), supporting that SNCA may serve as an additional biomarker for determining poor cognitive functions (19). TGFB1 is an important anti-inflammatory cytokine. It has been shown that its lower production may predict a longitudinal functional and cognitive decline in oldest-old individuals (20). Cumulatively, these results strongly suggest that one of the mechanisms by which LAP alleviates adenine-induced cognitive impairment may *via* modulating SNCA- and/or TGFB1- mediated neuroinflammation. Further research on studying how LAP may alter SNCA and/or TGFB1 expression and function is warranted.

LAP is a chain of nine lactate molecules, it is likely that LAP is broken to lactate and then lactate affect gene expression pattern and cognitive functions. Lactate has long been considered a byproduct of glycolysis (glucose breakdown) in anaerobic metabolism and a product of oxygen-limited metabolism. However, subsequent studies revealed that lactate is formed under both aerobic and anaerobic conditions (21). It was shown that lactate is taken up by liver and is converted to pyruvate by LDH to be used in Krebs cycle. Nevertheless, other studies proposed the lactate shuttle hypothesis and demonstrated that lactate generated in peripheral tissue is transferred to other tissues to be used as fuel. For instance, lactate produced in muscles (during physical exercise) could be taken by heart and other tissues (e.g., brain) and used as fuel (22, 23). Therefore, it is also likely that LAP exerts its effects though these mechanisms.

Our study showed that IMB significantly alleviated adenine-induced cognitive impairment. It is also possible that reduction of inflammation/neuroinflammation may be one of the important mechanisms. IMB, to the less extend than LAP, reduced inflammatory lesions in kidney tissues, circulating levels of proinflammatory cytokines, and mRNA levels of proinflammatory cytokines in kidney tissues (13). In this study, we found that IMB significantly reversed adenine-induced alterations of mRNA levels of ELOVL2, GLUT4, Nestin, TGFB1, and TGFB2 genes. ELOVL2 improves synaptic functionality and regulates/mitigates brain inflammatory activity (24). It also inhibits apoptosis in pancreatic beta cells supporting its involvement in metabolic processes (25). GLUT4 is an insulin-regulated glucose transporter. Animal models of insulin resistance showed memory deficit and a decrease in GLUT4 and hippocampal insulin signaling (26). The reduced GLUT4 expression in brain tissues of adenine-treated mice indicated that cognitive impairment is associated with impaired glucose utilization/energy metabolism in brain. The reduced GLUT4 expression in brain tissues of adenine-treated mice indicated impaired neural stem cell production and reduced neurogenesis in brain. While the primary function of Nestin is related to neurogenesis from neural stem cells, neuroinflammation is also involved in Nestin-mediated neurogenesis (27). The significantly reduced Nestin expression in adenine-treated mice indicated impaired neurogenesis, which could be reversed by IMB treatment. In addition to TGFB1, TGFB2 also has anti-inflammatory functions (28). Similar to TGFB1, the reduced expression level of TGFB2 in adenine-treated mice was significantly increased by IMB. These cumulative experimental results strongly suggest that IMB may improve cognition in part *via* inhibiting neuroinflammation, increasing energy metabolism, and increasing neurogenesis processes.

IMB was prepared by a koji fermentation of defatted soybeans with *Aspergillus oryzae* and lactic acid bacteria (*Pediococcus parvulus* and *Enterococcus faecium*), followed by water extraction and purification of koji polysaccharides. Hydrolysis analysis showed that this polysaccharide was mainly consisted with arabinose (41.4%), galactose (23.7%), and xylose (10.4%) (13). The functional roles of polysaccharides in modulating gut microbiota have been well-established. In this study, our microbiome analysis revealed that adenine treatment drastically altered the microbiome profile of the fecal samples of mice cecum. In fact, the abundance of almost 2/3 of bacterial species was changed at a significant level. Interestingly, IMB (especially IMB-H), but not LAP, could normalize the abundance of 30% of the altered bacteria.

We found that mice in the MC had significantly increased abundance of *Actinobacteria* phylum and significantly decreased abundance of *Bacteroidetes* phylum. Previous research has suggested that *Actinobacteria* (mainly Bifidobacterium) may play a functional role in cognition. Patients with cognitive impairment had a higher abundance of *Actinobacteria* than that of the controls (29). Mice with depression also had increased abundance of *Actinobacteria* (30). Ovariectomized (OVX) mice fed a high-fat diet experienced impaired object recognition and spatial memory associated with increased *Bifidobacteriales*; administration of epigallocatechin gallate (EGCG) significantly improved cognition and memory and inhibited the increase of *Bifidobacteriales* (31). The results in our current study showed that IMB supplementation significantly improved cognition associated with significant reduction of *Actinobacteria* abundance. Further analysis also showed that some *Bifidobacterium* species in the *Actinobacteria* phylum, such as the common bacteria *s*_*Bifidobacterium_pseudolongum* (Figure 6B), and the very rare bacteria s*_Bifidobacterium_*sp.*_AGR2158* and *s_Bifidobacterium_animalis* (Figure 7B), had significantly increased abundances in MC mice, which were reversed/normalized by IMB supplementation. These results suggest that IMB may improve cognition in part *via* inhibition of the abundance/function of bacteria species in *Actinobacteria* phylum.

Our results showed that the abundance of *Bacteroidetes* phylum (which includes notably *Bacteroides* and *Prevotella* genera) was significantly decreased in mice of adenine-induced cognitive impairment, and IMB treatment improved cognition associated with increased abundance of *Bacteroidetes*. It is well recognized that cognitive impairment is correlated to decreased abundance of *Bacteroidetes* and the increased *Firmicutes*/*Bacteroidetes* ratio. Alzheimer’s disease patients had significantly increased fecal abundance of *Firmicutes* but decreased abundance of *Bacteroidetes* compared to normal controls (32). Fecal microbiota transplantation improved cognition in patients with cognitive decline associated with enrichment of *Bacteroidetes* (33). Administration of young blood plasma significantly diminished the gut *Firmicutes/Bacteroidetes* ratio in middle-aged rats (34). Again, these results suggest that IMB may improve cognition in part *via* inhibition of the function of bacteria species in *Bacteroidetes* phylum.

**Figure 8:**
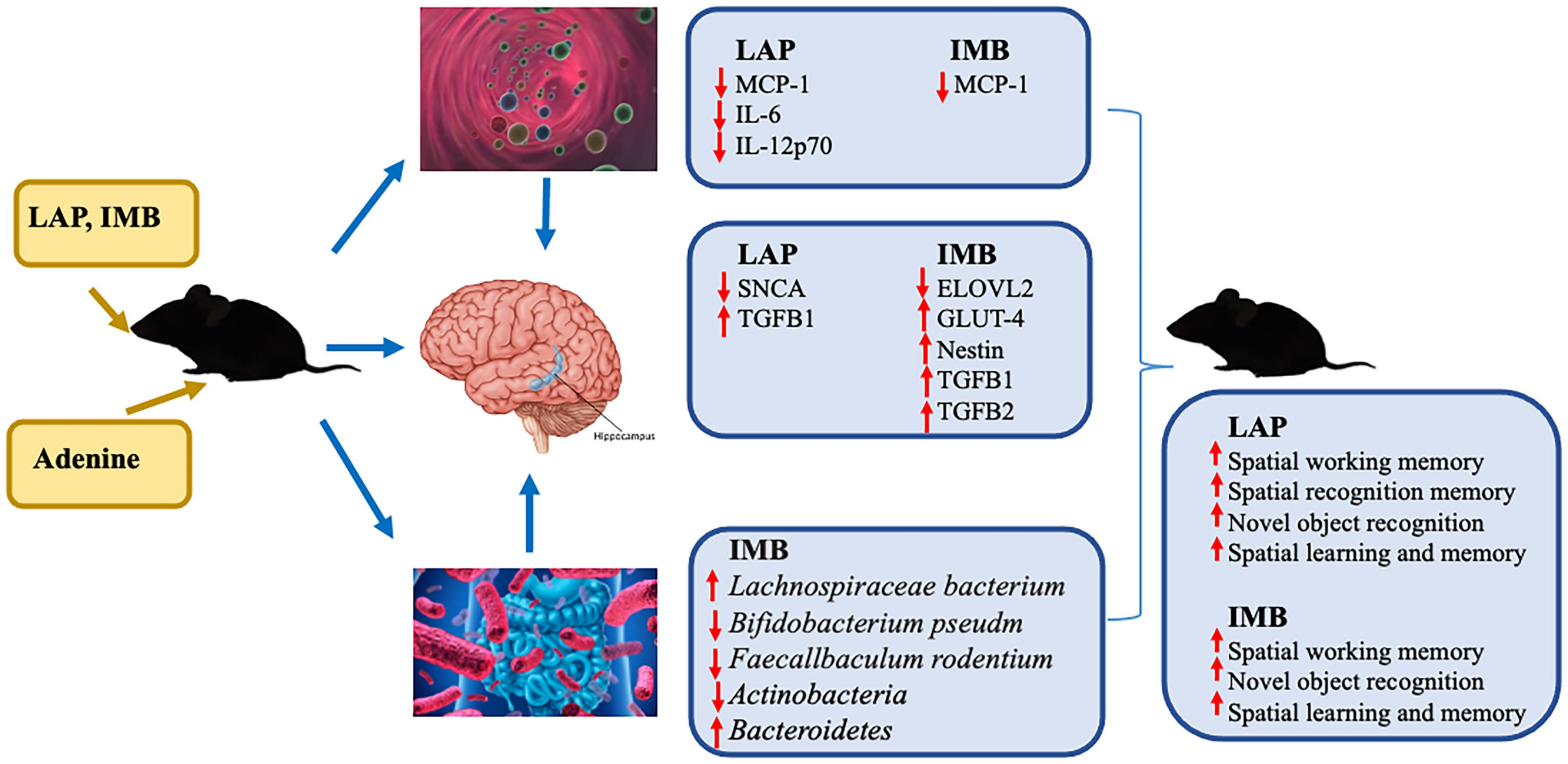
A diagram of suggested mechanisms of IMB and LAP actions. The results of circulating inflammatory cytokines are from our previous study (13).

In conclusion, our data demonstrated that LAP and IMB could improve cognitive performance in mice *via* distinct mechanisms of action. LAP may improve cognition in part *via* inhibiting inflammation/neuroinflammation and modulating metabolic process, whereas IMB may improve cognition in part *via* inhibiting inflammation/neuroinflammation, enhancing energy metabolism, increasing neurogenesis, and modulating gut microbiota (Figure 8).

## Data availability statement

The data presented in the study are deposited in the Harvard Dataverse, accession number (https://doi.org/10.7910/DVN/XUJYVB).

## Ethics statement

The animal study was reviewed and approved by Beth Israel Deaconess Medical Center.

## Author contributions

HA and YS contributed to the animal study, sample analysis, and manuscript preparation. HA and J-RZ contributed to experimental design and data analyses. J-RZ contributed to supervision and project administration. All authors contributed to the article and approved the submitted version.

## Funding

This research was funded by Nichimo Biotics Co., Ltd., Japan and LifeTrade Co., Ltd., Japan. The funders were not involved in the study design, collection, analysis, interpretation of data, the writing of this article or the decision to submit it for publication.

## Conflict of interest

The authors declare that this study received funding from Nichimo Biotics Co., Ltd and LifeTrade Co., Ltd. The funders were not involved in the study design, collection, analysis, interpretation of data, the writing of this article or the decision to submit it for publication.

## Publisher’s note

All claims expressed in this article are solely those of the authors and do not necessarily represent those of their affiliated organizations, or those of the publisher, the editors and the reviewers. Any product that may be evaluated in this article, or claim that may be made by its manufacturer, is not guaranteed or endorsed by the publisher.

## Footnotes

^1^ www.corebiome.com
